# 2-Amino-4,6-dimethyl­pyrimidine–anthranilic acid (1/1)

**DOI:** 10.1107/S1600536810003661

**Published:** 2010-02-03

**Authors:** Samuel Ebenezer, Packianathan Thomas Muthiah

**Affiliations:** a School of Chemistry, Bharathidasan University, Tiruchirappalli 620 024, Tamilnadu, India

## Abstract

In the title 1:1 adduct, C_6_H_9_N_3_·C_7_H_7_NO_2_, the crystal structure is stabilized by hydrogen bonds involving two different *R*_2_^2^(8) motifs. One of them is formed by the inter­action of 2-amino-4,6-dimethyl­pyrimidine (AMPY) with the carboxyl group of anthranilic acid (AA) through N—H⋯O and O—H⋯N hydrogen bonds, whereas the other is formed through the inter­action of two centrosymmetrically related pyrimidines involving N—H⋯N hydrogen bonds. These two combined motifs form a heterotetra­mer. The heterotetra­mer sheets are stacked into three-dimensional network.

## Related literature

For the importance the reaction of amino­pyrimidine derivatives and carboxylic acids in protein–nucleic acid recognition and drug binding, see: Hunt *et al.* (1980 ▶); Baker & Santi (1965 ▶). For pyrimidine–carboxylic acid inter­actions, see: Allen *et al.* (1999 ▶). For co-crystals of AMPY, see: Balasubramani *et al.* (2005 ▶, 2006 ▶); Devi & Muthiah (2007 ▶). For hydrogen-bonded synthons, see: Thakur & Desiraju (2008 ▶). For packing patterns in 2-amino-4,6-dimethyl­pyrimidine-salicylate, see: Muthiah *et al.* (2006 ▶). For typical geometric parameters in aromatic stacking, see: Hunter (1994 ▶).
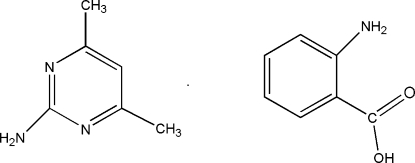


## Experimental

### 

#### Crystal data

C_6_H_9_N_3_·C_7_H_7_NO_2_
*M* *_r_* = 260.30
Triclinic, 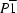

*a* = 7.1922 (2) Å
*b* = 7.4269 (2) Å
*c* = 13.0675 (3) Å
α = 77.583 (1)°
β = 78.990 (1)°
γ = 82.473 (1)°
*V* = 666.19 (3) Å^3^
*Z* = 2
Mo *K*α radiation
μ = 0.09 mm^−1^
*T* = 293 K
0.28 × 0.22 × 0.20 mm

#### Data collection

Bruker SMART APEXII CCD area-detector diffractometer
Absorption correction: multi-scan (*SADABS*; Bruker, 2008 ▶) *T*_min_ = 0.975, *T*_max_ = 0.982
16171 measured reflections
4279 independent reflections
3021 reflections with *I* > 2σ(*I*)
*R*_int_ = 0.024

#### Refinement

*R*[*F* ^2^ > 2σ(*F* ^2^)] = 0.053
*wR*(*F* ^2^) = 0.174
*S* = 1.04
4279 reflections
182 parameters
H atoms treated by a mixture of independent and constrained refinement
Δρ_max_ = 0.27 e Å^−3^
Δρ_min_ = −0.24 e Å^−3^

### 

Data collection: *APEX2* (Bruker, 2008 ▶); cell refinement: *SAINT* (Bruker, 2008 ▶); data reduction: *SAINT*; program(s) used to solve structure: *SHELXS97* (Sheldrick, 2008 ▶); program(s) used to refine structure: *SHELXL97* (Sheldrick, 2008 ▶); molecular graphics: *PLATON* (Spek, 2009 ▶); software used to prepare material for publication: *PLATON*.

## Supplementary Material

Crystal structure: contains datablocks global, I. DOI: 10.1107/S1600536810003661/kp2248sup1.cif

Structure factors: contains datablocks I. DOI: 10.1107/S1600536810003661/kp2248Isup2.hkl

Additional supplementary materials:  crystallographic information; 3D view; checkCIF report

## Figures and Tables

**Table 1:** Hydrogen-bond geometry (Å, °)

*D*—H⋯*A*	*D*—H	H⋯*A*	*D*⋯*A*	*D*—H⋯*A*
O1—H1⋯N1	0.81	1.90	2.7014 (13)	168
N2—H2*A*⋯N3^i^	0.86	2.26	3.0745 (14)	159
N2—H2*B*⋯O2	0.86	1.98	2.8303 (15)	169
N4—H4*A*⋯O2	0.94 (2)	1.91 (2)	2.6571 (17)	135.5 (17)
N4—H4*B*⋯N4^ii^	0.89 (2)	2.62 (2)	3.1409 (18)	118.7 (17)

Symmetry codes: (i) 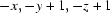; (ii) 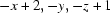.

**Fig. 1.**
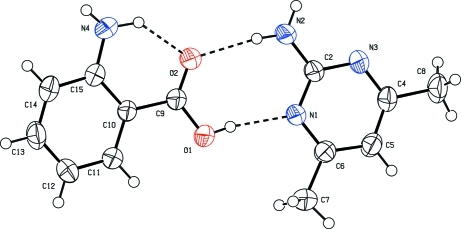
ORTEP view of the title compound showing the 50% probability displacement ellipsoids. Dashed lines indicate hydrogen bonds.

**Fig. 2.**
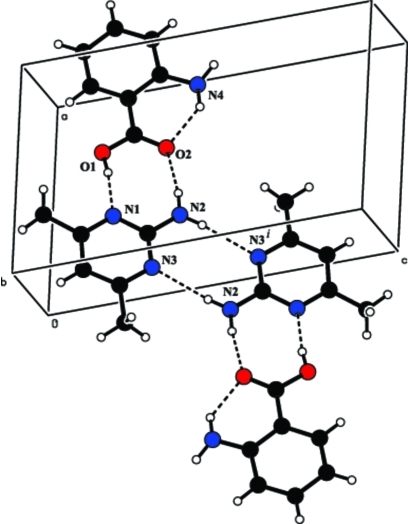
View of a heterotetrameric synthon formed through the O—H···N, N—H···O and N—H···N hydrogen bonds [Symmetry codes: (i) -x,1-y,1-z].

**Fig. 3.**
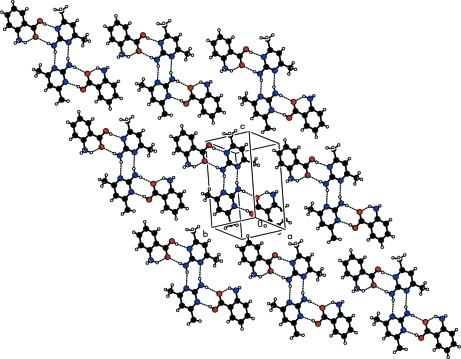
View of heterotetrameric synthons arranged parallel to the (122) plane.

**Fig. 4.**
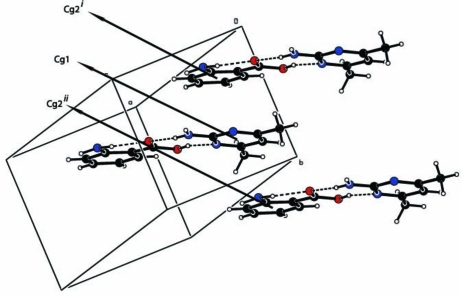
Stacking interactions observed between the pyrimidine ring and the aryl ring. Cg1 and Cg2 represents the centroid of the pyrimidine ring and aryl ring respectively [Symmetry codes: (i) -1+x,y,z ] (ii) -1+x,1+y,z].

## supplementary crystallographic information

### Comment

The aminopyrimidine derivatives, in nature as components of nucleic acids, and drugs are relevant for biological functions. Their interactions with carboxylic acids are of utmost importance since they are involved in protein –nucleic acid recognition and drug binding (Hunt *et al.*,1980; Baker & Santi, 1965). In general aminopyrimidines posses self complementary hydrogen-bonded motifs forming a base pair which in itself is an unique property. In addition, aminopyrimidines readily form pyrimidine-carboxylic acid interaction with ease which is evident from the survey carried out by Allen *et al.* (1999).

The present study has been focused on the analyses of the hydrogen bonding patterns in the cocrystal of 2-amino-4,6-dimethylpyrimidine-anthranilic acid. Several cocrystals of AMPY such as 2-amino-4,6-dimethylpyrimidine-cinnamicacid (1:2), 2-amino-4,6-dimethylpyrimidine-4-hydroxybenzoic acid (1/1) and 2-amino-4,6-dimethylpyrimidine-terephthalicacid have been reported from our laboratory (Balasubramani *et al.*, 2005; Balasubramani *et al.*, 2006; Devi & Muthiah, 2007).

The asymmetric unit contains a molecule of AMPY and AA (Fig. 1). The N1 and the amino group of AMPY interact with the carboxyl group of AA via O—H···N and N—H···O hydrogen bonds (Table 1) generating ring motif with graph set notation R_2_^2^(8). In addition, another type of R_2_^2^(8) motif is formed by centrosmmetrically related pyrimidine molecules through a pair of N—H···N hydrogen bonds. These two different motifs generate a linear heterotetrameric unit (Fig. 2) known to be one of the most stable synthons (Thakur & Desiraju, 2008).

One can expect similarity between the overall packing patterns of the title complex and 2-amino-4,6-dimethylpyrimidine-salicylate salt which has been earlier reported from our laboratory (Muthiah *et al.*, 2006). The primary level of organization is similar in both structures as they form a linear heterotetrameric synthon. However, the planarity of the heterotetraameric synthons is different. The heterotetrameric synthon formed in AMPY-AA is planar whereas that of 2-amino-4,6-dimethylpyrimidinium salicylate salt is not planar. In the title complex  heterotetramers are arranged as sheets (Fig. 3) which are stabilized through stacking interactions. The same is also observed in the aminopyrimidine salicylate salt with two different types of sheets arranged alternatively one over the other.

AMPY forms stacking interactions with aromatic rings (Fig. 4) of the 2ABA molecules above and below its plane with perpendicular separations of 3.468 and 3.624 %A, respectively; centroid-to-centroid distances of 3.641 (7) and 3.934 (7)%A, offset distances of 1.130 and 1.858 %A  and slip angles of 18.06 and 28.62, respectively. These geometric parameters are typical of aromatic stacking values (Hunter, 1994).

### Experimental

A hot methanolic solution (20 ml) of 2-amino-4,6-dimethylpyrimidine (Aldrich) and anthranilic acid (Loba Chemie)in the ratio 1:1  was warmed for 0.5 h over a water bath. The mixture was cooled slowly and kept at room temperature and after a few days, colourless crystals were obtained.

### Refinement

The hydrogen atoms of the N4 (H4A, H4B) were located in difference Fourier map and refined freely. The other hydrogen atoms were positioned geometrically and were refined using a riding mode. The C—H and O—H bond lengths are 0.93–0.96 and 0.82Å respectively [*U*iso(H)= 1.2Ueq (C, O)].

### Crystal data

**Table:**

C_6_H_9_N_3_·C_7_H_7_NO_2_	*Z* = 2
*M**_r_* = 260.30	*F*(000) = 276
Triclinic, *P*1	*D*_x_ = 1.298 Mg m^−^^3^
Hall symbol: -P 1	Mo *K*α radiation, λ = 0.71073 Å
*a* = 7.1922 (2) Å	Cell parameters from 4279 reflections
*b* = 7.4269 (2) Å	θ = 1.6–31.3°
*c* = 13.0675 (3) Å	µ = 0.09 mm^−^^1^
α = 77.583 (1)°	*T* = 293 K
β = 78.990 (1)°	Prism, brown
γ = 82.473 (1)°	0.28 × 0.22 × 0.20 mm
*V* = 666.19 (3) Å^3^	

### Data collection

**Table:**

Bruker SMART APEXII CCD area-detector diffractometer	4279 independent reflections
Radiation source: fine-focus sealed tube	3021 reflections with *I* > 2σ(*I*)
graphite	*R*_int_ = 0.024
φ and ω scans	θ_max_ = 31.3°, θ_min_ = 1.6°
Absorption correction: multi-scan (*SADABS*; Bruker, 2008)	*h* = −10→10
*T*_min_ = 0.975, *T*_max_ = 0.982	*k* = −10→10
16171 measured reflections	*l* = −19→19

### Refinement

**Table:**

Refinement on *F*^2^	Primary atom site location: structure-invariant direct methods
Least-squares matrix: full	Secondary atom site location: difference Fourier map
*R*[*F*^2^ > 2σ(*F*^2^)] = 0.053	Hydrogen site location: inferred from neighbouring sites
*wR*(*F*^2^) = 0.174	H atoms treated by a mixture of independent and constrained refinement
*S* = 1.04	*w* = 1/[σ^2^(*F*_o_^2^) + (0.0991*P*)^2^ + 0.0554*P*] where *P* = (*F*_o_^2^ + 2*F*_c_^2^)/3
4279 reflections	(Δ/σ)_max_ < 0.001
182 parameters	Δρ_max_ = 0.27 e Å^−^^3^
0 restraints	Δρ_min_ = −0.24 e Å^−^^3^

### Special details

**Table:**

Geometry. Bond distances, angles etc. have been calculated using the rounded fractional coordinates. All su's are estimated from the variances of the (full) variance-covariance matrix. The cell esds are taken into account in the estimation of distances, angles and torsion angles
Refinement. Refinement on *F*^2^ for ALL reflections except those flagged by the user for potential systematic errors. Weighted *R*-factors wR and all goodnesses of fit *S* are based on *F*^2^, conventional *R*-factors *R* are based on *F*, with *F* set to zero for negative *F*^2^. The observed criterion of *F*^2^ > σ(*F*^2^) is used only for calculating -*R*-factor-obs etc. and is not relevant to the choice of reflections for refinement. *R*-factors based on *F*^2^ are statistically about twice as large as those based on *F*, and *R*-factors based on ALL data will be even larger.

### Fractional atomic coordinates and isotropic or equivalent isotropic displacement parameters (Å^2^)

**Table:**

	*x*	*y*	*z*	*U*_iso_*/*U*_eq_	
N1	0.27302 (13)	0.63647 (13)	0.24381 (7)	0.0389 (3)	
N2	0.24994 (14)	0.50645 (17)	0.42161 (8)	0.0546 (4)	
N3	−0.01789 (13)	0.67010 (14)	0.36590 (8)	0.0438 (3)	
C2	0.16646 (15)	0.60562 (16)	0.34206 (9)	0.0398 (3)	
C4	−0.09918 (16)	0.77159 (16)	0.28620 (10)	0.0428 (3)	
C5	−0.00014 (17)	0.80839 (17)	0.18345 (10)	0.0465 (4)	
C6	0.18851 (16)	0.73870 (15)	0.16483 (9)	0.0406 (3)	
C7	0.3091 (2)	0.7734 (2)	0.05754 (10)	0.0551 (4)	
C8	−0.30286 (18)	0.8452 (2)	0.31434 (13)	0.0589 (4)	
O1	0.62794 (12)	0.47921 (13)	0.18713 (7)	0.0520 (3)	
O2	0.62400 (13)	0.35172 (16)	0.35704 (7)	0.0646 (3)	
N4	0.94272 (18)	0.17934 (18)	0.41965 (9)	0.0555 (3)	
C9	0.70902 (15)	0.38005 (16)	0.26562 (9)	0.0402 (3)	
C10	0.90843 (15)	0.30730 (14)	0.23577 (8)	0.0364 (3)	
C11	0.99344 (17)	0.33560 (16)	0.12857 (9)	0.0438 (3)	
C12	1.18091 (18)	0.27621 (19)	0.09731 (11)	0.0517 (4)	
C13	1.28729 (18)	0.18659 (19)	0.17468 (12)	0.0536 (4)	
C14	1.20848 (18)	0.15583 (18)	0.27979 (11)	0.0503 (4)	
C15	1.01624 (16)	0.21446 (15)	0.31397 (9)	0.0408 (3)	
H2A	0.18650	0.48560	0.48510	0.0650*	
H2B	0.36720	0.46320	0.40940	0.0650*	
H5	−0.05890	0.87820	0.12830	0.0560*	
H7A	0.23640	0.85160	0.00780	0.0830*	
H7B	0.35080	0.65770	0.03500	0.0830*	
H7C	0.41780	0.83320	0.06070	0.0830*	
H8A	−0.36050	0.87650	0.25150	0.0710*	
H8B	−0.30820	0.95370	0.34400	0.0710*	
H8C	−0.37030	0.75250	0.36550	0.0710*	
H1	0.51810	0.51100	0.20940	0.0780*	
H4A	0.820 (3)	0.238 (3)	0.4358 (16)	0.088 (6)*	
H4B	1.032 (3)	0.151 (3)	0.4607 (16)	0.093 (6)*	
H11	0.92120	0.39610	0.07720	0.0530*	
H12	1.23520	0.29570	0.02580	0.0620*	
H13	1.41450	0.14680	0.15460	0.0640*	
H14	1.28300	0.09490	0.32990	0.0600*	

### Atomic displacement parameters (Å^2^)

**Table:**

	*U*^11^	*U*^22^	*U*^33^	*U*^12^	*U*^13^	*U*^23^
N1	0.0312 (4)	0.0465 (5)	0.0358 (5)	0.0002 (4)	−0.0026 (3)	−0.0059 (4)
N2	0.0341 (5)	0.0840 (8)	0.0344 (5)	0.0112 (5)	−0.0005 (4)	−0.0018 (5)
N3	0.0305 (4)	0.0512 (5)	0.0450 (5)	0.0019 (4)	−0.0010 (4)	−0.0073 (4)
C2	0.0306 (5)	0.0491 (6)	0.0368 (5)	0.0006 (4)	−0.0025 (4)	−0.0073 (4)
C4	0.0325 (5)	0.0406 (5)	0.0537 (7)	0.0012 (4)	−0.0077 (5)	−0.0084 (5)
C5	0.0404 (6)	0.0455 (6)	0.0501 (7)	0.0025 (5)	−0.0125 (5)	−0.0016 (5)
C6	0.0387 (5)	0.0410 (5)	0.0403 (6)	−0.0029 (4)	−0.0069 (4)	−0.0043 (4)
C7	0.0544 (7)	0.0623 (8)	0.0406 (6)	−0.0004 (6)	−0.0029 (5)	0.0001 (5)
C8	0.0343 (6)	0.0600 (8)	0.0762 (9)	0.0086 (5)	−0.0082 (6)	−0.0089 (7)
O1	0.0348 (4)	0.0706 (6)	0.0412 (5)	0.0105 (4)	−0.0062 (3)	0.0000 (4)
O2	0.0427 (5)	0.0916 (7)	0.0414 (5)	0.0189 (5)	0.0008 (4)	0.0043 (4)
N4	0.0513 (6)	0.0679 (7)	0.0395 (5)	0.0100 (5)	−0.0121 (5)	0.0012 (5)
C9	0.0341 (5)	0.0442 (6)	0.0384 (5)	0.0016 (4)	−0.0052 (4)	−0.0037 (4)
C10	0.0319 (5)	0.0371 (5)	0.0372 (5)	0.0010 (4)	−0.0053 (4)	−0.0040 (4)
C11	0.0395 (6)	0.0476 (6)	0.0392 (6)	0.0032 (5)	−0.0053 (5)	−0.0032 (4)
C12	0.0421 (6)	0.0576 (7)	0.0483 (7)	0.0027 (5)	0.0037 (5)	−0.0094 (5)
C13	0.0353 (6)	0.0558 (7)	0.0657 (8)	0.0078 (5)	−0.0039 (5)	−0.0142 (6)
C14	0.0400 (6)	0.0522 (7)	0.0571 (7)	0.0081 (5)	−0.0152 (5)	−0.0082 (5)
C15	0.0392 (5)	0.0387 (5)	0.0428 (6)	0.0024 (4)	−0.0100 (4)	−0.0054 (4)

### Geometric parameters (Å, °)

**Table:**

O1—C9	1.3136 (15)	C7—H7A	0.9600
O2—C9	1.2214 (14)	C7—H7C	0.9600
O1—H1	0.8100	C7—H7B	0.9600
N1—C6	1.3390 (15)	C8—H8C	0.9600
N1—C2	1.3535 (14)	C8—H8B	0.9600
N2—C2	1.3330 (16)	C8—H8A	0.9600
N3—C4	1.3320 (16)	C9—C10	1.4748 (16)
N3—C2	1.3507 (15)	C10—C15	1.4073 (16)
N2—H2B	0.8600	C10—C11	1.4001 (15)
N2—H2A	0.8600	C11—C12	1.3750 (18)
N4—C15	1.3627 (16)	C12—C13	1.387 (2)
N4—H4A	0.94 (2)	C13—C14	1.364 (2)
N4—H4B	0.89 (2)	C14—C15	1.4110 (18)
C4—C8	1.5003 (18)	C11—H11	0.9300
C4—C5	1.3830 (18)	C12—H12	0.9300
C5—C6	1.3828 (17)	C13—H13	0.9300
C6—C7	1.4910 (17)	C14—H14	0.9300
C5—H5	0.9300		
			
O1···C7	3.3882 (17)	C9···H2B	2.8800
O1···N1	2.7014 (13)	C11···H11^viii^	2.9900
O2···N4	2.6571 (17)	C12···H11^viii^	3.0700
O2···N2	2.8303 (15)	H1···C7	2.9500
O1···H11	2.4000	H1···H2B	2.6000
O2···H2B	1.9800	H1···C6	2.8100
O2···H4A	1.91 (2)	H1···N1	1.9000
N1···O1	2.7014 (13)	H1···C2	2.8800
N2···N3^i^	3.0745 (14)	H2A···H4A^iii^	2.4800
N2···O2	2.8303 (15)	H2A···N3^i^	2.2600
N3···N2^i^	3.0745 (14)	H2A···C2^i^	3.1000
N4···N4^ii^	3.1409 (18)	H2B···C9	2.8800
N4···O2	2.6571 (17)	H2B···H1	2.6000
N1···H1	1.9000	H2B···O2	1.9800
N2···H4A^iii^	2.87 (2)	H4A···H2A^iii^	2.4800
N3···H2A^i^	2.2600	H4A···O2	1.91 (2)
N3···H4B^iii^	2.84 (2)	H4A···C9	2.48 (2)
N4···H4B^ii^	2.62 (2)	H4A···N2^iii^	2.87 (2)
C2···C15^iv^	3.3428 (16)	H4B···N4^ii^	2.62 (2)
C2···C14^iv^	3.5670 (18)	H4B···H4B^ii^	2.32 (3)
C4···C9^iv^	3.4584 (17)	H4B···N3^iii^	2.84 (2)
C6···C13^v^	3.5249 (18)	H4B···H14	2.3000
C7···O1	3.3882 (17)	H5···H7A	2.4000
C9···C4^vi^	3.4584 (17)	H5···H8A	2.4400
C13···C6^vii^	3.5249 (18)	H7A···H5	2.4000
C14···C2^vi^	3.5670 (18)	H8A···H5	2.4400
C15···C2^vi^	3.3428 (16)	H11···O1	2.4000
C2···H1	2.8800	H11···C11^viii^	2.9900
C2···H2A^i^	3.1000	H11···C12^viii^	3.0700
C6···H1	2.8100	H11···H11^viii^	2.4500
C7···H13^viii^	3.0800	H13···C7^viii^	3.0800
C7···H1	2.9500	H14···H4B	2.3000
C9···H4A	2.48 (2)		
			
C9—O1—H1	110.00	C4—C8—H8B	109.00
C2—N1—C6	117.21 (10)	C4—C8—H8C	109.00
C2—N3—C4	116.98 (10)	H8A—C8—H8B	109.00
C2—N2—H2A	120.00	C4—C8—H8A	109.00
C2—N2—H2B	120.00	H8A—C8—H8C	109.00
H2A—N2—H2B	120.00	H8B—C8—H8C	109.00
H4A—N4—H4B	127.5 (19)	O2—C9—C10	122.73 (11)
C15—N4—H4B	112.9 (13)	O1—C9—O2	121.75 (11)
C15—N4—H4A	113.4 (12)	O1—C9—C10	115.52 (10)
N1—C2—N3	124.84 (10)	C9—C10—C15	120.80 (10)
N1—C2—N2	117.74 (10)	C11—C10—C15	119.51 (10)
N2—C2—N3	117.42 (10)	C9—C10—C11	119.66 (10)
N3—C4—C8	116.34 (11)	C10—C11—C12	121.70 (11)
N3—C4—C5	121.65 (11)	C11—C12—C13	118.63 (12)
C5—C4—C8	122.01 (12)	C12—C13—C14	121.14 (13)
C4—C5—C6	118.28 (11)	C13—C14—C15	121.38 (12)
N1—C6—C7	116.38 (11)	N4—C15—C14	119.47 (11)
C5—C6—C7	122.59 (11)	C10—C15—C14	117.64 (11)
N1—C6—C5	121.03 (11)	N4—C15—C10	122.90 (11)
C6—C5—H5	121.00	C10—C11—H11	119.00
C4—C5—H5	121.00	C12—C11—H11	119.00
C6—C7—H7B	109.00	C11—C12—H12	121.00
C6—C7—H7C	109.00	C13—C12—H12	121.00
C6—C7—H7A	109.00	C12—C13—H13	119.00
H7A—C7—H7B	109.00	C14—C13—H13	119.00
H7A—C7—H7C	109.00	C13—C14—H14	119.00
H7B—C7—H7C	109.00	C15—C14—H14	119.00
			
C6—N1—C2—N2	−178.98 (11)	O2—C9—C10—C11	176.12 (12)
C6—N1—C2—N3	0.06 (17)	O2—C9—C10—C15	−5.78 (18)
C2—N1—C6—C5	−0.37 (17)	C9—C10—C11—C12	177.60 (12)
C2—N1—C6—C7	179.13 (11)	C15—C10—C11—C12	−0.53 (18)
C4—N3—C2—N1	−0.10 (18)	C9—C10—C15—N4	2.90 (18)
C4—N3—C2—N2	178.93 (11)	C9—C10—C15—C14	−177.28 (11)
C2—N3—C4—C5	0.47 (17)	C11—C10—C15—N4	−178.99 (12)
C2—N3—C4—C8	−178.87 (11)	C11—C10—C15—C14	0.83 (16)
N3—C4—C5—C6	−0.78 (19)	C10—C11—C12—C13	−0.2 (2)
C8—C4—C5—C6	178.53 (12)	C11—C12—C13—C14	0.6 (2)
C4—C5—C6—N1	0.72 (18)	C12—C13—C14—C15	−0.3 (2)
C4—C5—C6—C7	−178.75 (12)	C13—C14—C15—N4	179.37 (13)
O1—C9—C10—C11	−4.29 (16)	C13—C14—C15—C10	−0.45 (19)
O1—C9—C10—C15	173.81 (10)		

Symmetry codes: (i) −*x*, −*y*+1, −*z*+1; (ii) −*x*+2, −*y*, −*z*+1; (iii) −*x*+1, −*y*+1, −*z*+1; (iv) *x*−1, *y*, *z*; (v) *x*−1, *y*+1, *z*; (vi) *x*+1, *y*, *z*; (vii) *x*+1, *y*−1, *z*; (viii) −*x*+2, −*y*+1, −*z*.

### Hydrogen-bond geometry (Å, °)

**Table:**

*D*—H···*A*	*D*—H	H···*A*	*D*···*A*	*D*—H···*A*
O1—H1···N1	0.81	1.90	2.7014 (13)	168
N2—H2A···N3^i^	0.86	2.26	3.0745 (14)	159
N2—H2B···O2	0.86	1.98	2.8303 (15)	169
N4—H4A···O2	0.94 (2)	1.91 (2)	2.6571 (17)	135.5 (17)
N4—H4B···N4^ii^	0.89 (2)	2.62 (2)	3.1409 (18)	118.7 (17)
C11—H11···O1	0.93	2.40	2.7353 (15)	101

Symmetry codes: (i) −*x*, −*y*+1, −*z*+1; (ii) −*x*+2, −*y*, −*z*+1.
